# Low-dose ferric carboxymaltose vs. oral iron for improving hemoglobin levels in postpartum East Asian women: A randomized controlled trial

**DOI:** 10.1371/journal.pone.0319795

**Published:** 2025-03-12

**Authors:** Takeshi Nagao, Ken Takahashi, Sho Takahashi, Ryo Yokomizo, Osamu Samura, Aikou Okamoto

**Affiliations:** 1 Department of Obstetrics and Gynecology, The Jikei University School of Medicine, Tokyo, Japan; 2 Center for Research Promotion, The Jikei University School of Medicine, Tokyo, Japan; Kasr Alainy Medical School, Cairo University, EGYPT

## Abstract

Ferric carboxymaltose (FCM) is widely used to correct anemia and replenish iron stores rapidly, particularly in Western populations. However, lower doses of FCM are typically used in East Asia, with limited research on their effectiveness, especially in postpartum women. This randomized controlled trial aimed to assess the efficacy of low-dose FCM compared with oral ferrous sulfate in increasing postpartum hemoglobin (Hb) levels and replenishing iron stores in East Asian women. Sixty postpartum women with Hb levels < 10 g/dL and serum ferritin ≤ 30 ng/mL were randomized to receive either intravenous FCM (500 mg at baseline and 2 weeks) or oral ferrous sulfate (210 mg daily for 4 weeks). The primary outcome was the increase in Hb levels at 2 weeks post-enrollment. Secondary outcomes included serum ferritin, transferrin saturation, the Edinburgh Postnatal Depression Scale (EPDS) score, and adverse events at 4 weeks. The FCM group demonstrated a significantly greater increase in Hb levels at 2 weeks (mean difference 0.42 g/dL; 95% CI: 0.12–0.72; P =  0.006), with markedly higher ferritin (adjusted mean difference 356.0 ng/mL; 95% CI: 321.0–403.0; P <  0.001) and transferrin saturation (adjusted mean difference 10.76%; 95% CI: 4.20–17.31; P =  0.002) at 4 weeks. Although there was no significant difference in final Hb levels at 4 weeks (mean difference 0.36 g/dL; 95% CI: -0.01–0.72; P =  0.055), the FCM group had a lower median EPDS score (median difference -3.0; 95% CI: -5.0 to -1.0; P =  0.002) and fewer gastrointestinal side effects, including constipation and nausea. Hypophosphatemia occurred asymptomatically in three patients in the FCM group. These findings suggest that low-dose FCM infusion is highly effective in increasing Hb levels at 2 weeks post-enrollment, with fewer gastrointestinal side effects and higher ferritin levels observed at 4 weeks post-enrollment compared with oral ferrous sulfate.

This study was registered at the UMIN Clinical Trials Registry, which meets the requirements of the ICMJE, on December 1, 2021 (ID: UMIN000046049).

## Introduction

Iron deficiency anemia (IDA) is a prevalent issue, affecting around 1-2 billion people globally, with postpartum women being particularly at risk [1]. The World Health Organization (WHO) defines anemia as hemoglobin (Hb) levels less than 10 g/dL, and reports from Europe indicate that approximately 50% of postpartum women meet this criterion [2–3]. This condition is often accompanied by decreased iron stores, which can exacerbate its negative effects. IDA has been identified as a risk factor for postpartum depression, anxiety, blood transfusions due to postpartum hemorrhage, and delayed infant development [4–6]. Despite being a very common issue, there are currently no high-quality, evidence-based treatment protocols for postpartum IDA. Addressing this condition promptly and effectively is crucial for improving maternal health outcomes.

The standard treatment for postpartum IDA typically involves oral iron supplementation due to its convenience and low cost [7]. However, oral iron therapy is often associated with gastrointestinal side effects, poor compliance, and slow replenishment of iron stores [8–9]. In cases where rapid correction of anemia is required, or when oral iron is ineffective or not tolerated, intravenous iron therapy is considered [7]. Traditional intravenous iron formulations, such as iron sucrose, require multiple administrations and carry risks of allergic reactions [10].

Ferric carboxymaltose (FCM) is a newer intravenous iron formulation that allows for the administration of larger single doses with a favorable safety profile [11]. Studies conducted in Western populations have demonstrated that FCM can achieve faster correction of anemia and better maintenance of iron stores compared to oral iron or other intravenous formulations [12–14]. For instance, trials have shown that single doses of 1,000 mg FCM significantly improved Hb levels and iron parameters more effectively than oral iron over a similar treatment period [12–14]. These studies underscore the potential benefits of FCM in managing postpartum IDA more efficiently.

In East Asia, including Japan, the context of postpartum IDA management differs from that in Western countries. As of January 2022, high-dose intravenous iron preparations such as ferumoxytol and ferric derisomaltose have not been approved in Japan, limiting the available options for intravenous iron therapy. Additionally, there is a tendency to use lower doses of intravenous iron in East Asian clinical practice compared to Western countries [15]. Specifically, while Western studies often utilize single high-dose administrations of 1,000 mg FCM, in East Asia, lower-dose regimens are more common. For example, a total dose of 1,000 mg FCM may be administered as two separate infusions of 500 mg each (2 ×  500 mg) [15]. This approach reflects regional practices and regulatory approvals. The distinction between low-dose (e.g., 2 ×  500 mg) and high-dose (e.g., single 1,000 mg) intravenous iron administration is important, as it may influence treatment efficacy and safety outcomes. While both dosing strategies aim to deliver the same total amount of iron, the differences in administration schedules could affect the rate of Hb increase, iron stores replenishment, and potential side effects.

This study aimed to evaluate the efficacy of FCM infusion in East Asian women with postpartum IDA, focusing on whether lower doses can effectively induce Hb increase and replenish iron stores. By addressing this gap, the study seeks to inform postpartum IDA treatment strategies in East Asia, ensuring they are both effective and tailored to the specific needs of this population.

## Materials and methods

### Study design

This open-label, single-center, randomized controlled trial was conducted at Jikei University Hospital, a high-volume tertiary care center in Tokyo, Japan. This study was conducted in accordance with the CONSORT checklist (S1 File).

### Participants

Patient recruitment was conducted from January 10 to September 10, 2022. All patients provided written informed consent to participate in the study. Patients with postpartum IDA (Hb < 10 g/dL, as defined by the WHO guideline for moderate to severe anemia, and serum ferritin ≤  30 ng/mL) were enrolled [2]. To minimize the influence of inflammation on the improvement of IDA, only patients with CRP levels ≤ 20 mg/L were included. For vaginal deliveries, laboratory assessments for enrollment were conducted on postpartum day 3, as patients typically remain hospitalized at that time. For cesarean sections, laboratory tests for enrollment were routinely performed on the first postoperative day to assess anemia. The timing of these laboratory assessments was determined according to standard postpartum care practices in Japan. Eligibility criteria require participants to be aged 20 or older and to possess the capacity for autonomous decision-making. Women with multiple pregnancies were excluded because their postpartum recovery and iron requirements may differ significantly from those of singleton pregnancies [16]. Patients with a history of psychiatric disorders (postpartum depression, major depression, anxiety disorder, or panic disorder) were excluded, as such conditions could potentially influence the evaluation of postpartum mental health. Other exclusion criteria included body weight <  35 kg at the enrollment of the study (as 500 mg doses are only approved for patients ≥ 35 kg in Japan) [15]; a history of any malignancy; a history of hematologic disorders, or severe hepatic, renal, or cardiovascular disorders (grade 3 or higher in the Common Terminology Criteria for Adverse Events version 5.0 of the National Cancer Institute) [17]. These conditions were excluded to minimize confounding factors that could influence Hb levels, anemia status, or iron metabolism. Additionally, the prescribing information for intravenous ferric carboxymaltose in Japan advises cautious administration in patients with these conditions due to the potential risk of adverse effects, further supporting their exclusion [15]. Other exclusion criteria included new-onset active vaginal bleeding (such as secondary postpartum hemorrhage) after study enrolment; and a history of severe drug allergies (such as anaphylactic shock) were excluded.

### Protocol

Participants were evaluated for signs of hypersensitivity or a history of adverse reactions to iron products before each FCM infusion. In the FCM group, 500 mg of FCM diluted in 100 mL saline was administered intravenously over 10 minutes at study enrollment and again at 2 weeks post-enrollment (Ferinject; Zeria Pharmaceutical Co., Japan) [15]. Patients were instructed not to take any additional iron supplements after discharge. The infusions were performed by registered nurses trained in intravenous therapy and emergency response, under the direct supervision of an attending physician. All infusions were conducted in a monitored clinical setting equipped with emergency equipment, including resuscitation devices and medications such as epinephrine and antihistamines. Patients were observed closely during the infusion and for at least 15 minutes afterward for signs of adverse reactions. To ensure participant safety, a predefined protocol was followed to manage any adverse events, which included the immediate cessation of the infusion and initiation of appropriate medical interventions. All adverse events were documented and reported to the institutional review board (IRB) in accordance with ethical guidelines.

Patients in the ferrous sulfate group were instructed to take two tablets of the supplement once daily after the evening meal (Fero-Gradumet; Mylan EPD, Japan) for 4 weeks from the start of the study. One tablet of ferrous sulfate contains 105 mg of elemental iron. This dosage was selected based on standard clinical practice in Japan for treating moderate to severe iron-deficiency anemia in postpartum women [18]. The patients were instructed not to take any iron supplements other than ferrous sulfate after discharge.

Data collection was conducted at multiple time points: at study enrollment (baseline), at 2 weeks post-enrollment (11–17 days after study enrollment), and at 4 weeks post-enrollment (25–31 days after study enrollment). Baseline data included demographic information, medical history, delivery details, and initial laboratory values. Laboratory tests were repeated at 2 weeks and 4 weeks post-enrollment to assess iron status, liver and kidney function, and electrolyte abnormalities. In the FCM group, laboratory tests at 2 weeks post-enrollment were conducted prior to the second iron infusion, with the results made available to both healthcare providers and participants to monitor for any significant adverse effects from the initial FCM infusion. However, the decision to proceed with the second infusion was not based on Hb or ferritin levels from these tests.

Blood tests for enrollment were conducted as laboratory tests, with venous blood samples collected while participants were lying down in the postpartum ward. Follow-up care was provided by nurses and attending physicians during scheduled outpatient visits at Jikei University Hospital. Blood tests at 2 weeks and 4 weeks post-enrollment were also conducted as laboratory tests, with samples collected at the hospital’s outpatient clinic while participants were seated. All samples were analyzed in the hospital’s central laboratory using standardized equipment and protocols to ensure consistency and accuracy.

The Edinburgh Postnatal Depression Scale (EPDS), a globally used screening tool for predicting postpartum depression onset, was administered at study enrollment and 4 weeks post-enrollment. In Japan, the EPDS is typically assessed at 4 weeks postpartum, aligning with standard clinical practice that emphasizes early detection and intervention for postpartum depression. A score of 9 or higher at this time point is considered indicative of a high risk for developing postpartum depression in Japanese studies [19].

At the time of study completion or withdrawal, all patients completed a questionnaire administered by research nurses. The questionnaire gathered information on any adverse events experienced, including constipation, diarrhea, nausea, erythema, headache, dysgeusia, or abdominal pain. Additionally, patients in the ferrous sulfate group who completed the study were asked at 4 weeks post-enrollment to report the number of days they missed taking ferrous sulfate, enabling researchers to assess adherence.

All data were collected by a single researcher who was not involved in the clinical care of the patients and entered into a secure electronic database using de-identified participant codes. A separate monitoring team was responsible for overseeing data integrity, and a dedicated statistician conducted the data analysis, ensuring objectivity throughout the process. Data management adhered to institutional guidelines for clinical research, including regular audits and quality checks to ensure accuracy and transparency (S2 File).

### Outcomes

The primary outcome was the increase in Hb levels from baseline to 2 weeks post-enrollment, measured in grams per deciliter. This time point was selected to assess the rapid efficacy of FCM compared to oral ferrous sulfate in increasing Hb levels during immediate postpartum recovery. Continuous Hb change was selected as the primary endpoint instead of a binary outcome such as IDA resolution because the former provides a more sensitive measure of treatment response and requires a feasible sample size, while still addressing comprehensive IDA management through secondary outcomes. Hb levels were measured using a multi-item automated hematology analyzer, the XN-10 by Sysmex Corporation.

Secondary outcomes were measured at 4 weeks post-enrollment, after the completion of the treatment course. These outcomes included:

Serum ferritin levels at 4 weeks post-enrollment, measured in nanograms per milliliter, to evaluate iron stores. Serum ferritin levels were quantified using the LABOSPECT 008α automatic analyzer by Hitachi High-Tech Corporation.

Serum Hb levels at 4 weeks post-enrollment,were measured using the same methods as those used at 2 weeks post-enrollment.

Transferrin saturation (TSAT) at 4 weeks post-enrollment, calculated as (serum iron/ total iron-binding capacity) ×  100%, to assess iron utilization. Normal TSAT values range from 30% to 50% in pregnant women [20].

EPDS scores at 4 weeks post-enrollment, a validated 10-item questionnaire for postpartum depression, with a scoring range of 0 to 30.

The incidence of adverse events was evaluated through laboratory tests and structured questionnaires. These questionnaires included standardized items addressing symptoms such as constipation, diarrhea, nausea, skin lesions (e.g., rash or hives), itching, headache, taste disturbances, and upper abdominal pain.

### Relevant definitions

Blood Loss Quantification: For vaginal deliveries, the total blood loss was calculated by measuring the weight of gauze and sheets used during the delivery process. For cesarean deliveries, blood loss was determined by combining the weight of used gauze and the volume recorded by the suction device. In both vaginal and cesarean deliveries, the measured blood loss may include amniotic fluid.

Gestational Age Determination: Gestational age was determined using the most precise information available. When the embryo transfer date or a clearly identified ovulation date was known, gestational age was calculated based on this information. In cases where such data was unavailable, gestational age was estimated using the last menstrual period (LMP) and the usual menstrual cycle. If the gestational age estimated by fetal ultrasound differed by more than 7 days from the LMP-based estimate, the gestational age derived from the ultrasound measurement was used as the final determination.

### Randomization

Eligible patients were randomly assigned in a 1:1 ratio to either the FCM group or the ferrous sulfate group using stratified block randomization with a block size of 4. Stratification was performed by delivery mode (vaginal delivery or cesarean section) and anemia severity (Hb level < 8 g/dL or ≥ 8 g/dL) to ensure balanced group allocation within each stratum. The randomization process was conducted using a web-based application to generate the stratified block randomization table [21]. The allocation sequence was securely stored within the application and managed by an independent researcher who was not involved in patient recruitment or clinical care. Allocation concealment was maintained by restricting access to the allocation sequence. The attending physicians and participants remained unaware of the assignment until informed consent was obtained, at which point the independent researcher communicated the group assignment to the study staff. As this was an open-label randomized controlled trial due to the nature of the intervention, no masking was applied.

### Statistical analysis

A sample size of 60 patients was determined to be appropriate for this study based on the results of a previous analysis [22]. As no previous study used 500 mg of FCM, we referred to a study in which ferrous sucrose was administered. Therefore, we assumed that the FCM group would exhibit a 1.6 g/dL mean increase in Hb level and the ferrous sulfate group would exhibit a 0.6 g/dL mean increase in Hb level [22]. Assuming a group difference of 1.0 g/dL and standard deviation of 1.0 g/dL, which was based on data from previous studies with similar populations, 23 patients per arm, with an equal number in each group, would provide > 90% power to detect a difference in Hb level reduction between the FCM and ferrous sulfate groups using a two-sided, two-sample *t*-test at a 5% level of significance and assuming no correlation between baseline and 2 weeks. Thus, assuming a dropout rate of approximately 23%, 30 participants were required per group, necessitating 60 participants in the study. A dropout rate of 23% was assumed due to the COVID-19 pandemic and the unique challenges faced by the postpartum population, such as fatigue and difficulty attending follow-up visits.

This study employed the Full Analysis Set (FAS) approach for analysis. The FAS was defined as the largest analysis population consisting of all participants who were enrolled in the study, randomized, received at least one dose of the study or control drug, had baseline laboratory results obtained on the enrollment day, and had no major protocol violations, such as lack of consent or enrollment outside the designated period.

For the baseline variables, summary statistics were constructed using frequency and proportion for categorical data and mean and standard deviation, or median and range (interquartile range or min-max), for continuous variables. For the primary analysis, the mean difference in Hb level reduction between the FCM and ferrous sulfate groups at 2 weeks post-enrollment and its 95% confidence interval (CI) were estimated using a linear mixed-effects model. The model included treatment group, time as a categorical variable and interaction between treatment and week, and adjusted for stratification factors (delivery mode and anemia severity) and baseline Hb level. An unstructured covariance structure was assumed. The secondary analysis of Hb level increase at 4 weeks post-enrollment was performed in the same manner as the primary analysis. For TSAT, an analysis of covariance model adjusted for stratification factors (delivery mode and anemia severity) and baseline outcome was performed. For serum ferritin level and the EPDS score, the Wilcoxon rank-sum test and Hodges–Lehmann estimator were used to determine the difference between the study groups. The proportion of patients with iron deficiency (ferritin ≤ 30 ng/mL) and EPDS score ≥ 9 was compared using Fisher’s exact test.

Safety analysis was performed to evaluate abnormal clinical laboratory values and the frequency and proportion of patient-reported adverse events. For the laboratory test at 4 weeks, continuous variables such as phosphorus levels and liver function tests were analyzed using Student’s two-sample t-test. Additionally, the proportion of patients with hypophosphatemia (<2.4 mg/dL) was compared using Fisher’s exact test, as the expected cell counts were less than five. For Patient Complaints, the frequency of reported symptoms such as constipation, diarrhea, nausea, and upper abdominal pain was compared between groups using Pearson’s chi-square test. When 20% or more of the cells had expected counts less than five, Fisher’s exact test was applied to ensure appropriate statistical evaluation.

All *P*-values were two-sided. No adjustment for multiple testing was made for secondary efficacy outcomes. Statistical significance was set at *P* <  0.05. All statistical analyses were performed using SAS version 9.4 (SAS Institute, Cary, NC, USA).

### Ethics statement

This study was registered at the UMIN Clinical Trials Registry on December 1, 2021 (ID UMIN000046049). It was approved by the Institutional Review Board of Jikei University Hospital (Tokyo, Japan) (approval no. 33-176-10793; September 24, 2021) (S3 File).

## Results

### Trial population

Sixty patients with postpartum IDA were randomly assigned to the FCM (n =  30) or ferrous sulfate group (n =  30). Although this study targeted patients with moderate to severe IDA as defined by the WHO, no participants presented with severe IDA, and all were classified as having moderate anemia (7.0 g/dL ≤  Hb ≤  9.9 g/dL) [2]. Baseline Hb levels were comparable in both groups (8.9 ±  0.7 g/dL in each group), with 16.7% of participants in each group presenting with Hb <  8 g/dL. Serum ferritin levels were slightly higher in the FCM group (21.5 ng/mL [IQR 16.0–25.0]) compared to the ferrous sulfate group (16.5 ng/mL [IQR 11.0–22.0]), but the proportion of patients with severe low ferritin (<15 ng/mL) was higher in the ferrous sulfate group (36.6%) than in the FCM group (23.3%) (Table 1).

**Table 1:** Baseline characteristics of the intravenous ferric carboxymaltose and oral ferrous sulfate groups.

Variable	Ferric carboxymaltose n = 30	Ferrous sulfate n = 30
**Maternal characteristic**		
Age, mean ± SD (years)	35.6 ± 4.0	34.9 ± 5.2
BMI, mean ± SD (kg/m²)	23.5 ± 3.0	23.9 ± 3.9
EPDS score, median (IQR)	2.5 (1.0–6.0)	3.0 (2.0–7.0)
EPDS score ≥ 9, n (%)	4 (13.3)	4 (13.3)
**Delivery characteristic**		
Gestation week, median (IQR)	38.0 (38.0–39.0)	38.0 (38.0–39.0)
Quantification of blood loss, median (IQR) (mL)	700 (615-960)	682 (536-900)
Cesarean section, n (%)	17 (56.7)	17 (56.7)
**Baseline laboratory test** (mean ± SD)		
Hb, mean ± SD (g/dL)	8.9 ± 0.7	8.9 ± 0.7
Hb < 8 g/dL, n (%)	5 (16.7)	5 (16.7)
Ferritin, median (IQR) (ng/mL)	21.5 (16.0-25.0)	16.5 (11.0-22.0)
Severe Low Ferritin (<15 ng/mL), n (%)	7 (23.3)	11 (36.6)
Transferrin saturation, mean ± SD (%)	14.4 ± 9.2	15.3 ± 9.7
Reticulocyte, mean ± SD (%)	2.3 ± 0.4	2.4 ± 0.5
Hematocrit, mean ± SD (%)	28.3 ± 2.4	28.8 ± 2.7
Mean corpuscular volume, mean ± SD (fL)	87.1 ± 5.6	87.5 ± 6.5
Mean corpuscular hemoglobin, mean ± SD (pg)	27.4 ± 2.4	27.6 ± 2.7
Folic acid, mean ± SD (ng/mL)	6.2 ± 3.4	5.9 ± 2.7
Vitamin B12, mean ± SD (pg/mL)	232.8 ± 83.9	236.3 ± 92.8
Aspartate aminotransferase, mean ± SD (IU/L)	25.4 ± 9.6	24.3 ± 9.5
Alanine aminotransferase, mean ± SD (IU/L)	16.1 ± 11.1	15.6 ± 13.6
γ-Glutamyl transpeptidase, mean ± SD (IU/L)	11.6 ± 12.4	11.4 ± 16.3
Phosphorus, mean ± SD (mg/dL)	3.9 ± 0.5	3.8 ± 0.5

BMI, body mass index; EPDS, Edinburgh Postnatal Depression Scale; Hb, hemoglobin; SD, standard deviation

This study employed the Full Analysis Set (FAS) approach for analysis. The FAS was defined as the largest analysis population consisting of all participants who were enrolled in the study, randomized, received at least one dose of the study or control drug, had baseline laboratory results obtained on the enrollment day, and had no major protocol violations, such as lack of consent or enrollment outside the designated period.

**Fig 1:**
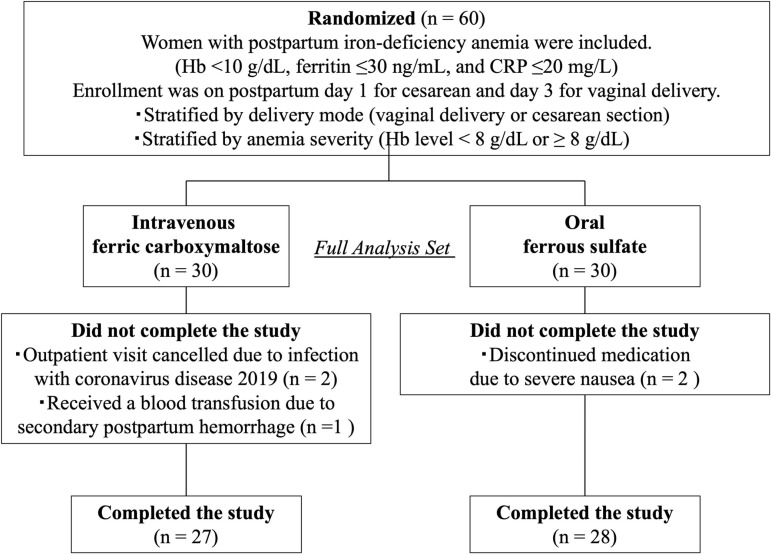
Study population. East Asian patients with postpartum iron-deficiency anemia were randomized to intravenous ferric carboxymaltose or oral ferrous sulfate groups, stratified by mode of delivery and anemia severity.

Of the 60 randomized patients, three in the FCM group were excluded due to study withdrawal: two canceled their outpatient visits after being diagnosed with COVID-19, and one received a blood transfusion for secondary postpartum hemorrhage. In the ferrous sulfate group, two patients withdrew due to severe nausea. As a result, baseline and safety analyses included all 60 randomized patients, while primary and secondary outcome analyses and laboratory safety assessments were performed on 27 participants in the ferric carboxymaltose group and 28 in the ferrous sulfate group (Fig 1).

### Primary outcome

The mean increase in Hb level from baseline to 2 weeks post-enrollment were higher in the FCM group than in the ferrous sulfate group (3.00 vs. 2.58 g/dL; adjusted mean difference =  0.42(95% CI =  0.11–0.72; P =  0.009); unadjusted mean difference =  0.43 (95% CI =  0.09–0.76)). At 2 weeks post-enrollment, anemia (Hb < 10 g/dL) was observed in one patient in each group; however, no patients in either group were identified as having iron deficiency anemia (ferritin ≤ 30 ng/mL, Hb < 10 g/dL) (Table 2). To address the potential collinearity between anemia severity and baseline Hb, we conducted additional analyses by excluding either anemia severity or baseline Hb from the model. These analyses confirmed that the results remained consistent and robust, indicating that collinearity did not significantly affect the findings.

**Table 2:** Comparison of Hemoglobin Increases and Secondary Outcomes Between Intravenous Ferric Carboxymaltose and Oral Ferrous Sulfate in Postpartum Women.

Variable	Ferric carboxymaltose n = 27	Ferrous sulfate n = 28	Adjusted mean difference (95% CI)	*P* *
**Primary outcome** (at 2 weeks)				
Hb increase, mean ± SD (g/dL)	3.00 ± 0.66	2.58 ± 0.58	0.42 (0.11–0.72)	**0.009**
Anemia (Hb < 10 g/dL), n (%)	1 (3.7)	1 (3.6)	–	–
Iron-deficiency anemia (Hb < 10 g/dL, ferritin ≤ 30 ng/mL), n (%)	0 (0)	0 (0)	–	–
**Secondary outcomes** (at 4 weeks)				
Ferritin, median (IQR) (ng/mL)	393.0 (331.0-472.0)	37.5 (26.0-52.0)	356.0 (321.0–403.0)	**<0.001**
Iron deficient (ferritin ≤ 30 ng/mL), n (%)	0 (0)	9 (32.1)	–	**0.002**
Hb increase, mean ± SD (g/dL)	3.79 ± 0.89	3.41 ± 0.77	0.36 (-0.01–0.72)	0.055
Anemia (Hb < 10 g/dL), n (%)	0 (0)	0 (0)	–	–
Iron-deficiency anemia (Hb < 10 g/dL, ferritin ≤ 30 ng/mL), n (%)	0 (0)	0 (0)	–	–
Transferrin saturation, mean ± SD (%)	39.5 ± 11.7	28.9 ± 12.0	10.76 (4.20–17.31)	**0.002**
EPDS score, median (IQR)	1.0 (0–16)	5.5 (0–13)	-3.0 (-5.0 to -1.0)	**0.002**
EPDS score ≥ 9, n (%)	2 (7.4)	6 (21.4)	–	0.252

Hb, hemoglobin; EPDS, Edinburgh Postnatal Depression Scale; SD, standard deviation.

Three patients in the intravenous ferric carboxymaltose group and two patients in the ferrous sulfate group withdrew from the study before the 2-week postpartum evaluation.

*For the primary analysis and the secondary analysis of Hb levels, a linear mixed-effects model was used. For transferrin saturation, an analysis of covariance model adjusted for stratification factors (delivery mode and anemia severity) and baseline outcome was performed. For serum ferritin level and the EPDS score, the Wilcoxon rank-sum test and Hodges–Lehmann estimator were used to determine the difference between the study groups. The proportion of patients with iron deficiency (ferritin ≤ 30 ng/mL) and EPDS score ≥ 9 was compared using Fisher’s exact test.

### Secondary outcomes

Secondary outcomes are summarized in Table 2. The median serum ferritin level at 4 weeks was higher in the FCM group than in the ferrous sulfate group (393.0 vs. 37.5 ng/mL; adjusted mean difference =  356.0; 95% CI =  321.0–403.0; *P* <  0.001). Nine patients in the ferrous sulfate group were iron deficient even after 4 weeks of oral administration, whereas this was not observed in the FCM group (0% vs. 32.1%; *P* =  0.002). At 4 weeks post-enrollment, no patients in either group were identified as having anemia (Hb < 10 g/dL) or iron deficiency anemia (ferritin ≤ 30 ng/mL, Hb < 10 g/dL). No significant difference was noted in the Hb increase at 4 weeks post-enrollment between the groups (3.79 vs. 3.41 g/dL; adjusted mean difference =  0.36; 95% CI =  -0.01 to 0.72; *P* =  0.055). TSAT at 4 weeks was higher in the FCM group than in the ferrous sulfate group (39.5 vs. 28.9%; adjusted mean difference =  10.76; 95% CI =  4.20–17.31; *P* =  0.002) (Table 2). The median EPDS score at 4 weeks was lower in the FCM group than in the ferrous sulfate group (1.0 vs. 5.5; Hodges–Lehmann estimate =  -3.0; 95% CI =  -5.0 to -1.0; *P* =  0.002). No significant difference was observed in the percentage of patients with suspected postpartum depression (EPDS ≥  9) between the FCM group and the ferrous sulfate group (7.4% vs. 21.4%; P =  0.252). The study was not powered to detect differences in this secondary outcome due to the limited sample size.

### Adverse events

No woman in either group experienced anaphylactic reactions, and none of the patients experienced extravasation or pigmentation following FCM infusion. Additionally, the laboratory tests performed at 4 weeks post-enrollment showed that no cases in either group had Alanine aminotransferase, Aspartate aminotransferase, or γ-Glutamyl transpeptidase levels exceeding more than twice the upper limit of normal. However, the serum phosphorus level was significantly lower in the FCM group compared to the ferrous sulfate group (3.5 vs. 4.2 g/dL; P <  0.001). In the FCM group, two patients were found to have hypophosphatemia (phosphate levels below 2.4 mg/dL [0.78 mmol/L]) at 4 weeks post-enrollment (7.4% vs. 0%; *P* =  0.028). All cases of hypophosphatemia in the FCM group were asymptomatic. In contrast, the number of patients with symptoms of constipation and nausea was higher in the ferrous sulfate group (33.3% in the FCM group vs. 63.3% in the ferrous sulfate group; *P* =  0.020 and 0% vs. 23.3%; *P* =  0.011, respectively) (Table 3). Additionally, there were no significant differences between the two groups in other adverse events such as diarrhea (10.0% in both groups; *P* =  1.000), erythema (3.3% in the FCM group vs. 13.3% in the ferrous sulfate group; *P* =  0.353), headache (10.0% in the FCM group vs. 26.7% in the ferrous sulfate group; *P* =  0.095), and upper abdominal pain (10.0% in the FCM group vs. 3.3% in the ferrous sulfate group; *P* =  0.612) (Table 3).

**Table 3:** Adverse Events Associated with Intravenous Ferric Carboxymaltose vs. Oral Ferrous Sulfate in Postpartum Women.

Variable	Ferric carboxymaltose	Ferrous sulfate	*P* *
Any symptoms of anaphylaxis, n (%)	0	0	–
Extravasation or pigmentation after iron infusion, n (%)	0	–	–
**Laboratory test at 4 weeks**^†^, mean ± SD			
Aspartate aminotransferase (IU/L)	20.1 ± 4.9	29.5 ± 5.9	0.677
Alanine aminotransferase (IU/L)	22.4 ± 11.6	18.2 ± 11.0	0.175
γ-Glutamyl transpeptidase (IU/L)	20.6 ± 13.9	17.8 ± 10.9	0.406
Phosphorus (mg/dL)	3.5 ± 0.6	4.2 ± 0.5	**<0.001**
Hypophosphatemia (<2.4 mg/dL), n (%)	2 (7.4%)	0	**0.028**
**Patient Complaints**, n (%)			
Constipation	10 (33.3)	19 (63.3)	**0.020**
Constipation Requiring Treatment (Mg Tablets)	10 (33.3)	13 (43.3)	0.426
Diarrhea	3 (10.0)	3 (10.0)	1.000
Nausea	0	7 (23.3)	**0.011**
Erythema	1 (3.3)	4 (13.3)	0.353
Headache	3 (10.0)	8 (26.7)	0.095
Dysgeusia	0	0	–
Upper abdominal pain	3 (10.0)	1 (3.3)	0.612

Mg, magnesium; SD, standard deviation.

†The analysis was conducted based on the full analysis set. However, for the laboratory test at 4 weeks, five patients withdrew from the study and could not be evaluated, resulting in an analysis performed with 27 participants in the ferric carboxymaltose group and 28 participants in the ferrous sulfate group.

*Continuous variables were analyzed using Student’s two-sample t-test, and ordinal variables were analyzed using the Wilcoxon rank-sum test. The frequency of adverse events was compared using Pearson’s chi-square test, with Fisher’s exact test applied if 20% or more of the cells had expected counts less than five.

### Self-reported oral ferrous sulfate compliance

Medication compliance in the ferrous sulfate group was assessed based on the number of days participants missed taking their medication over the study period. Of the 28 participants, 14 (50%) reported no missed days, while 8 (29%) missed between 1 and 5 days. Three participants (11%) missed 6 to 10 days, and 2 participants (7%) missed 11 to 15 days. Only 1 participant (4%) missed 16 to 20 days, while no participants (0%) reported missing more than 20 days.

## Discussion

Our study demonstrated that low-dose FCM infusions significantly increased postpartum Hb levels more rapidly than oral ferrous sulfate after just one infusion, with a notably lower incidence of gastrointestinal adverse effects observed after two infusions. Additionally, no patients treated with low-dose FCM were identified as iron deficient at 4 weeks post-enrollment, whereas approximately one-third of patients in the ferrous sulfate group remained iron deficient, underscoring the clinical efficacy of low-dose FCM in managing postpartum IDA, particularly in East Asian patients. This study is among the first randomized controlled trials to evaluate the efficacy of low-dose FCM in an East Asian postpartum population, using regionally relevant doses of intravenous and oral iron to provide clinically applicable data. The strength of our study lies in its focus on a specific population and the use of a lower FCM dosage than previously studied, which still resulted in significant clinical improvements. These findings highlight the practical benefits of FCM, including improved adherence and reduced side effects, making it a valuable option for optimizing postpartum anemia treatment protocols and enhancing patient outcomes in East Asian populations.

Previous randomized controlled trials have consistently shown the superiority of FCM over oral iron in treating postpartum IDA, with faster Hb level improvements and better overall outcomes [23–25]. Notably, earlier studies administered higher FCM doses (1,000 mg or 15 mg/kg body weight) than our study. Our results corroborate these findings, demonstrating that even the lower FCM dose approved in East Asia is sufficient for rapid moderate IDA correction. Furthermore, our study highlights the importance of maintaining adequate ferritin levels, a goal less consistently achieved with oral iron therapy.

The rapid increase in Hb levels observed with FCM in our study underscores the importance of effective early intervention in postpartum moderate IDA. The primary goal in managing IDA is not only to normalize Hb levels but also to restore serum ferritin levels to ensure adequate iron stores. Achieving normal Hb levels signifies effective management of anemia, while attaining target ferritin levels, which represent stored iron, ensures sufficient iron reserves to support ongoing red blood cell production and reduce the risk of relapse. Intravenous iron therapy, including FCM, is effective but may temporarily increase labile plasma iron, raising concerns about oxidative stress [26]. While some studies in chronic kidney disease patients have shown that a single dose of intravenous iron does not trigger oxidative stress or inflammation biomarkers, the long-term safety, particularly in postpartum women, remains unclear and requires further research [27]. While lower doses of oral iron (80–120 mg/day) are effective for moderate to severe iron-deficiency anemia and are associated with fewer side effects and improved adherence, the higher doses commonly used in Japan may lead to gastrointestinal discomfort, potentially lowering adherence among postpartum women. In contrast, the reduced gastrointestinal side effects associated with FCM might have had the potential to influence adherence, reinforcing its role as a first-line treatment for postpartum anemia [28].

Secondary outcomes such as mental health were considered; however, factors affecting postpartum mental status, as previously reported, such as a family history of postpartum depression, marital status, educational level, and poor social and financial support during the puerperium, were not evaluated in this study. [29–32]. Additionally, as the sample size was calculated based on the primary outcome, the potential impact of the early increase in Hb levels with intravenous iron on mental health requires cautious interpretation. In this study, due to the sample size, we focused on comparing median EPDS scores, but future larger-scale studies are needed that adjust for these factors and analyze the proportion of participants at high risk for postpartum depression (EPDS score ≥ 9). Such studies would provide more definitive conclusions and meaningful insights into the potential impact on postpartum mental health.

This study has several limitations. Potential bias may have arisen from differences in enrollment timing. One cesarean section patient was enrolled on day 1, while others with vaginal deliveries were enrolled on day 3, reflecting common timing of laboratory tests in Japan. Since Hb and ferritin levels may change over these two days, there is a possibility that the severity of anemia was not assessed in the same way between cesarean section and vaginal delivery patients. However, as block randomization by delivery mode was used, this discrepancy was effectively mitigated, thereby reducing the potential for bias in our findings. Additionally, as this was an open-label study, performance and detection bias could have been introduced due to the lack of blinding. To address this, objective outcomes such as Hb and ferritin levels were used, and standardized protocols for data collection and laboratory assessments were applied to ensure consistency. Another limitation is that we did not monitor post-discharge diet and medications, which could have influenced the outcomes. In the ferrous sulfate group, adherence was assessed only through self-reported measures, which are prone to recall bias. However, the 50% non-adherence rate observed in this group suggests that the improved outcomes in the FCM group may stem not only from the superiority of the medication itself but also from the advantages of its administration method. Despite this, the clinical significance of FCM remains evident, particularly in reducing gastrointestinal side effects and avoiding issues related to adherence [11]. Additionally, the total amount of iron administered differed between the groups. By the time the primary outcome was evaluated, the FCM group had received 500 mg of iron intravenously, whereas the ferrous sulfate group had only been administered 420 mg (14 days ×  210 mg elemental iron =  2940 mg, with an absorption rate calculated at 14.3%) [33]. However, the dosages used are consistent with those approved and commonly prescribed in East Asia, enhancing the relevance of the findings for evaluating the real-world effectiveness of these treatments in clinical practice within the region [15,18].

In conclusion, FCM infusion, administered according to the East Asian protocol, is highly effective in increasing Hb levels at 2 weeks post-enrollment, with fewer gastrointestinal side effects and higher ferritin levels observed at 4 weeks post-enrollment compared with oral ferrous sulfate. The results support the use of FCM for rapid increases in Hb levels and iron stores replenishment in postpartum women, particularly in populations with similar demographic characteristics. These findings underscore the importance of integrating FCM into treatment protocols for postpartum anemia and highlight the need for further research to explore its long-term benefits in postpartum care.

## Supporting information

S1 File CONSORT Checklist. The CONSORT checklist for the study is provided in this file, detailing adherence to the CONSORT reporting guidelines.
(DOCX)

S2 File Study Protocol. This document provides the detailed study protocol, including the research design, methodology, and analysis plan used in the study.
(DOCX)

S3 File Ethics approval. This document contains the ethics approval for the study provided by the the Jikei University School of Medicine, including the reference number 33-176(10793).
(DOCX)
