# Supplementary material for: Low-dose ferric carboxymaltose vs. oral iron for improving hemoglobin levels in postpartum East Asian women: A randomized controlled trial
Source: PLoS One. 2025 Mar 12;20(3):e0319795. doi: 10.1371/journal.pone.0319795 (PMC11902287; doi:10.1371/journal.pone.0319795)
Supplement: S2 File — This document provides the detailed study protocol, including the research design, methodology, and analysis plan used in the study. (DOCX) [file pone.0319795.s002.docx]

**Study protocol (English)**

Primary and Secondary Outcome Measures

1.1 Primary Outcome Measure

Change in hemoglobin (Hb) levels at 2 weeks after administration (2 weeks postpartum).

• Calculation: Value at 2 weeks after administration − Value at 0 weeks on the registration and administration start date.

Rationale:

To evaluate the improvement in anemia.

1.2 Secondary Outcome Measures

(1) Change in ferritin levels, Hb levels, and transferrin saturation (TSAT = serum iron ÷ TIBC × 100 (%)) at 4 weeks after administration (4 weeks postpartum).

(2) Changes in the Edinburgh Postnatal Depression Scale (EPDS) scores.

Rationale:

(1) To evaluate the improvement in anemia.

(2) To examine the impact of anemia on postpartum depression.

1.3 Safety Outcome Measure

Incidence of adverse events.

Rationale:

Set as a general measure to evaluate safety.

2. Study Design

• Nature of the Study: Confirmatory

• Design: Parallel-group comparison

• Randomization: Randomized

• Blinding: Open-label (non-blinded)

• Control Group: Oral iron supplement treatment group

This study is a single-center randomized controlled trial targeting 60 postpartum women with iron deficiency anemia. To test the primary hypothesis, postpartum women with iron deficiency anemia (Hb <10 g/dL and ferritin <30 ng/mL) will be randomly assigned to receive either iron carboxymaltose injection or extended-release ferrous sulfate tablets.

Blinding is not possible due to the differences in treatment schedules between the iron carboxymaltose injection and extended-release ferrous sulfate tablets. Event evaluation will be conducted by a monitoring officer in a blinded manner using centralized assessment.

To verify the study hypothesis, the participation period for individual subjects is set at 1 month postpartum, referencing previous studies on postpartum iron deficiency anemia.

3. Patient Enrollment and Allocation

3.1 Patient Enrollment Method

The following procedure will be followed to promptly enroll patients after obtaining written consent:

(1) The principal investigator or researchers will obtain written consent from the subject and confirm eligibility.

(2) Eligible subjects will be assigned identification codes prior to study treatment initiation.

(3) Study treatment will be initiated according to allocation results.

3.2 Person in Charge of Patient Enrollment

• Institution: Jikei University Hospital

• Department: Obstetrics and Gynecology

• Address: 3-19-18 Nishi-Shinbashi, Minato-ku, Tokyo 105-0003

• Tel: 03-3433-1111

3.3 Allocation

• Allocation Ratio: 1:1

• Method: Stratified block randomization

• Adjustment Factors: Mode of delivery (vaginal delivery, cesarean section), severity of anemia (Hb < 8 g/dL, Hb ≥ 8 g/dL).

• Rationale for Adjustment Factors: To account for differences in blood loss during delivery and the effect of anemia treatment based on anemia severity.

3.4 Person in Charge of Patient Enrollment and Allocation: Takeshi Nagao

The person responsible for drug allocation will create an allocation table consisting of serial numbers, randomized orders of the control drug and active drug names (iron carboxymaltose injection or Ferro-Gradumet tablets), and study drug allocation codes.

4. Drugs (or Medical Devices) Used in the Study

4.1 Iron Carboxymaltose Injection

• Generic Name: Iron carboxymaltose injection

• Brand Name: Feinject Injection 500mg

• Approval Number: 23100AMX00290000

• Indications: Iron deficiency anemia

• Dosage: 500 mg administered intravenously once a week, maximum 1,500 mg total.

• Storage: Store at room temperature (avoid freezing).

4.2 Extended-release Ferrous Sulfate Tablets

• Generic Name: Extended-release ferrous sulfate tablets

• Brand Name: Ferro-Gradumet 105mg tablets

• Approval Number: 22100AMX01333000

• Indications: Iron deficiency anemia

• Dosage: 105–210 mg of iron daily, taken 1–2 times a day on an empty stomach.

• Storage: Store at room temperature.

5. Observation and Examination Items

5.1 Patient Background

Age, body weight at delivery, height, mode of delivery, blood loss during delivery, gestational age at delivery.

5.2 Subjective Symptoms and Objective Findings

Confirmed through questionnaires and visual inspection at each visit.

5.3 Observation of Adverse Events

All adverse events will be recorded, including severity, seriousness, causal relationship, onset time, outcome, and resolution time.

5.4 Vital Signs

Blood pressure, pulse rate, respiratory rate, body temperature.

5.5 Body Weight

Body weight and BMI.

5.6 Laboratory Tests

Blood tests will be conducted twice, requiring 10 ml of blood each time.

6. Target Number of Subjects and Planned Period

6.1 Target Number of Subjects

The target number of subjects is 60:

• Study drug group (iron carboxymaltose injection): 30 cases

• Control group (extended-release ferrous sulfate tablets): 30 cases

6.2 Study Period

• Study implementation period: From the date of Ethics Committee approval until March 31, 2025 (extensions will be made as necessary).

• Patient enrollment period: From the date of Ethics Committee approval until March 31, 2023.

**Statistical protocol**

1. Purpose of the Study

To evaluate the safety and efficacy of iron carboxymaltose injection compared to oral iron supplementation for the treatment of postpartum iron deficiency anemia.

1.1 Overview of the Study Design

Design: Randomized, open-label, standard treatment-controlled, parallel-group comparative study.

Groups:

Treatment Group: Iron carboxymaltose injection

Control Group: Extended-release ferrous sulfate tablets

1.2 Primary Outcome Measure

Efficacy:

Change in Hb levels at 2 weeks after administration (2 weeks postpartum).

Calculation: Change = Value at 2 weeks after administration − Value at baseline (registration and start of treatment).

1.3 Secondary Outcome Measures

Efficacy: (1) Change in ferritin levels, Hb levels, and transferrin saturation (TSAT = serum iron ÷ TIBC × 100 (%)) at 4 weeks after administration (4 weeks postpartum).

(2) Changes in the Edinburgh Postnatal Depression Scale (EPDS) scores.

Safety: (1) Incidence and frequency of adverse events.

2. General Considerations in Statistical Analysis

2.1 Interim Analysis

No interim analysis will be conducted in this study.

2.2 Data Monitoring

Data monitoring will be performed in accordance with the procedures specified in the protocol.

2.3 Data Handling

The analysis will target fixed data after the follow-up period ends.

For values below the detection limit, half of the detection limit value will be substituted.

Definitions for change and rate of change:

Change: Measurement after intervention − Baseline measurement.

Rate of Change: (Change / Baseline measurement) × 100 (%).

If necessary, variable transformations such as square root or logarithmic transformations will be performed.

Missing data will not be considered in the analysis or secondary analysis, but sensitivity analysis (e.g., multiple imputation) will be conducted as needed to assess the stability of the results.

2.4 Significance Level and Confidence Interval

Unless otherwise specified, the significance level for hypothesis testing will be two-sided at 5%, and 95% confidence intervals will be calculated.

2.5 Adjustment for Multiplicity

Unless otherwise specified, no adjustment for multiplicity will be performed.

2.6 Common Rules for Data Processing

Calculation of days and periods:

Unless otherwise specified, the number of days will be calculated by subtracting the start date from the end date and adding 1.

Example:

Duration of medication: If the start and end of treatment occur on the same day, the duration is counted as 1 day.

Time to adverse event: If the adverse event occurs the day after the start of treatment, the duration to the first adverse event is 2 days.

Survival period: If the patient dies the day after the start of treatment, the survival period is 2 days.

Conversion of days to years, months, weeks:

1 year = 365.25 days, 1 month = 30.4375 days, 1 week = 7 days.

Display of period or occurrence relative to the start of treatment:

The start date of treatment will be counted as day 1, and the day before the start date as day -1.

When the term “each time point” is used, it refers to all time points where data is available for analysis.

Decimal Places and Rounding Rules:

Means, standard deviations, and medians will be displayed to one decimal place (rounding at the second decimal place).

Percentages will be displayed to one decimal place (e.g., 12.3%), rounding at the second decimal place.

No rounding will occur during intermediate calculations; rounding will only be applied to final results.

2.7 Confidence Intervals for Proportions of Events

Confidence intervals for the proportion of occurrences or response rates will be calculated using the exact method based on the F-distribution.

3. Analysis Population

3.1 Definition of Analysis Populations

3.1.1 Full Analysis Set (FAS)

The FAS will include all subjects enrolled in the study, randomized, and who received at least one dose of the study or control drug, and for whom Hb test results from the baseline visit can be obtained. Subjects with major protocol violations (e.g., lack of consent or enrollment outside the designated period) will be excluded.

3.1.2 Per Protocol Set (PPS)

The PPS will include subjects from the FAS, excluding those with major violations of the protocol, such as:

Violation of inclusion/exclusion criteria

Use of prohibited concomitant medications or therapies.

3.1.3 Safety Analysis Set

The safety analysis will include all subjects who were randomized and received at least one dose of the study or control drug, grouped by the treatment actually received.

3.2 Correspondence with Statistical Analysis Items

Efficacy evaluations will primarily be based on the FAS. The PPS analysis will be conducted as a reference. Safety evaluations will be based on the safety analysis set.

4. Plan for Breakdown and Exposure Analysis

4.1 Breakdown of Subjects

The breakdown of randomized subjects will be presented.

4.2 Summary of Withdrawals or Discontinuations

A list of subjects who withdrew or discontinued will be provided.

5. Analysis Plan for Subject Background

Summary statistics of baseline characteristics will be calculated for each analysis population by group.

For categorical variables, the frequency and percentage will be shown. For continuous variables, summary statistics (sample size, mean, standard deviation, minimum, 1st quartile, median, 3rd quartile, and maximum) will be presented.

Between-group comparisons will be made using Pearson’s chi-square test for categorical variables (Fisher’s exact test if more than 20% of expected frequencies are below 5) and Student’s t-test or the Wilcoxon rank-sum test for continuous variables. A two-sided significance level of 5% will be used.

6. Analysis Plan for Primary Outcome

The primary objective of this study is to demonstrate the superiority of iron carboxymaltose injection over extended-release ferrous sulfate tablets in terms of the change in Hb levels at 2 weeks postpartum.

The primary outcome will be analyzed by comparing the mean change in Hb levels between the two groups. The null hypothesis is that the mean change in Hb for the treatment group is equal to that of the control group (mean change difference = 0), and the alternative hypothesis is that the mean change differs between the groups.

A two-sided 95% confidence interval for the mean change difference will be calculated. Covariate-adjusted ANCOVA will be used, adjusting for factors such as delivery mode (vaginal delivery or cesarean section), severity of anemia (Hb < 8 g/dL or Hb ≥ 8 g/dL), and baseline Hb levels.

7. Analysis Plan for Secondary Outcomes

To supplement the primary analysis, secondary outcomes will be analyzed without adjusting for multiplicity. A two-sided significance level of 5% and 95% confidence intervals will be calculated.

The change in ferritin, Hb, and TSAT levels at 4 weeks postpartum will be summarized by sample size, mean, standard deviation, minimum, 1st quartile, median, 3rd quartile, and maximum. Covariate-adjusted ANCOVA will be used to analyze these outcomes.

EPDS score progression will be evaluated by calculating the proportion of subjects with scores ≥ 9 at baseline and at 4 weeks postpartum. Between-group comparisons will be conducted using the chi-square test and adjusted Mantel-Haenszel test (risk difference) with covariates for delivery mode and severity of anemia.

8. Analysis Plan for Safety Outcomes

8.1 Safety Outcome Analysis Plan

The primary safety outcome is the frequency and proportion of adverse events. Adverse event data will be summarized by the number of cases and proportion in each group, with 95% exact binomial confidence intervals. Between-group comparisons will be made using Pearson’s chi-square test (Fisher’s exact test if needed).

8.2 Clinical Laboratory Data and Vital Signs

Summary statistics for clinical laboratory data and vital signs will be calculated for each group. Categorical variables will be compared using Pearson’s chi-square test, while continuous variables will be compared using Student’s t-test or the Wilcoxon rank-sum test.

9. Statistical Analysis Implementation

9.1 Responsible Statistician

Affiliation: Clinical Research Support Center, Jikei University School of Medicine

Name: Sho Takahashi

9.2 Statistical Software

Statistical analyses will be performed using SAS 9.4 or later (Windows version, 32-bit or 64-bit).
