# Supplementary material for: Low-dose ferric carboxymaltose vs. oral iron for improving hemoglobin levels in postpartum East Asian women: A randomized controlled trial
Source: PLoS One. 2025 Mar 12;20(3):e0319795. doi: 10.1371/journal.pone.0319795 (PMC11902287; doi:10.1371/journal.pone.0319795)
Supplement: S3 File — This document contains the ethics approval for the study provided by the the Jikei University School of Medicine, including the reference number 33-176(10793). (DOCX) [file pone.0319795.s003.docx]

**September 24, 2021**

**Ethics Committee Review Result Notification**

Department: Obstetrics and Gynecology
Applicant: Professor Aikou Okamoto
Tokyo Jikei University School of Medicine
President: Senya Matsufuji

Application number: 33-176 (10793)

**Research Title:**
A randomized superiority trial to examine the safety and efficacy of ferric carboxymaltose injection in postpartum iron deficiency anemia

Principal Investigator: Takeshi Nagao

As a result of the ethics committee review of the above application, we notify you of the following decision:

Review Date: September 6, 2021

**Approved**

2. Not Approved

3. Application Not Required

4. Requires Revision

- If the implementation facility is one of the affiliated hospitals, the necessary procedures should be followed at each affiliated hospital.
- Monitoring should be conducted in accordance with ethical guidelines.
